# Effect of Thymectomy on Outcomes of Myasthenia Gravis Patients: A Case-Control Study at a Tertiary Care Hospital

**DOI:** 10.7759/cureus.37584

**Published:** 2023-04-14

**Authors:** Imran Khawaja

**Affiliations:** 1 Department of Internal Medicine, Ayub Teaching Hospital, Abbottabad, PAK

**Keywords:** thymoma, pharmacological remission, complete remission, thymectomy, myasthenia gravis

## Abstract

Background and objective

Myasthenia gravis (MG) is an acquired autoimmune disease mediated by antibodies affecting the neuro-muscular junction on the postsynaptic membrane, resulting in neuromuscular transmission obstruction and, consequently, muscle weakening. It is believed that the thymus gland plays a critical role in the production of these antibodies. Screening patients for thymoma and surgical excision of the thymus gland is a crucial part of the treatment. To compare the odds of good outcomes in Myasthenia Gravis patients with or without thymectomy.

Material and methods

A retrospective case-control study was conducted at the Department of Medicine and Neurology, Ayub Teaching Hospital, Abbottabad, Pakistan, from October 2020 to September 2021. A purposive sampling technique was employed. Thirty-two MG patients with thymectomy and 64 MG patients without thymectomy were selected for investigation. Controls and cases were matched on the basis of sex and age (1:2). A positive EMG study, acetylcholine receptor antibodies, and a pyridostigmine test were used to make the diagnosis of MG. Patients were called to the outpatient department for assessment of treatment outcomes. Primary outcome evaluation was done using the Myasthenia Gravis Foundation of America Post-Intervention Status (MGFA-PIS) tool at the last follow-up after one year.

Results

A sample of 96 patients was evaluated, of which 63 (65%) were females and 33 (34%) were males. The mean age for Group 1 (cases) was 35 years ±8.9 and for Group 2 (controls) was 37± 11.1. Age and Osserman stages were shown to be the two most crucial prognostic factors in our study. However, there are several other factors in our study that are linked to a poor response, such as a greater BMI, dysphagia, thymoma, older age, and a longer duration of disease.

Conclusions

Our findings indicate that none of the analysed groups had significantly worse outcomes as a result of the current clinical practice of thymectomy patient selection.

## Introduction

Myasthenia gravis (MG) is an acquired autoimmune disease mediated by antibodies involving the neuro-muscular junction on the postsynaptic membrane, which results in blockage of neuromuscular transmission. MG is characterized by varying and frequently crippling muscular weakness. One in every 6500 is affected by this acquired disorder, and up to 10% of those affected by the illness might become treatment-resistant, which highlights the need for more efficient and individualized treatment approaches [1,2].

In the previous 50 years, population-based research on the epidemiology of the disease has been carried out. The reports of these studies suggest a definite rising trajectory. In the United States, recent estimates place the frequency at around 20 per 100,000 [3]. Today, it is understood that there are various subtypes of the illness, each with its own set of underlying pathological mechanisms, some of which are associated with the thymus gland. This is especially true for non-thymomatous MG linked to autoantibodies against the nicotinic acetylcholine receptor (AChR-Abs) and thymoma-associated MG (TAMG), where the thymus frequently exhibits inflammation [4]. Thymectomy has been used for a long time for both thymomatous and non-thymomatous MG, though its efficacy in the latter case is still debatable. After the pivotal Thymectomy Trial in Non-Thymomatous Myasthenia Gravis Patients Receiving Prednisone Therapy (MGTX) trial's release in 2016, which included patients with acetylcholine receptor antibody (AChR-Ab)-positive non-thymomatous MG under 65 years old, this debate was finally put to rest. After three years of follow-up, extended thymectomy was found to be more effective than therapeutic treatment alone in improving myasthenic symptoms and eliminating the need for hospital admissions and steroid therapy [5]. Thymectomy for MG is often safe and well tolerated, and in the majority of patients, it is associated with a persistent improvement in symptoms [6]. Furthermore, the impact followed through after five years in a smaller subset of patients who finished the MGTX extension study [7]. Although the favourable effects were clearly proven, the study was unable to ascertain if they would apply equally to all subgroups inside the study procedure or would also apply beyond. It is still unknown if variables such as the timing of the operation (up to five years after the onset of MG in the study), the type of thymectomy done, the gender of the patient, or the histology of the thymus might influence the outcomes.

Although it is unlikely that randomised studies with stringent inclusion criteria would ever find a solution to these issues, they remain highly important to clinical practice. In order to gain a deeper understanding of the influence of patient factors on surgical outcomes, it is crucial to review outcomes data in depth [8]. There is a dire need to fill the research gap on the present topic as thymectomy is a common practice for MG and data on its potential benefits have rarely been reported in Pakistan. This study was intended to help us better understand the conceivable advantages of thymectomy for MG patients. Additionally, the objective of this study was to compare the clinical outcomes of patients who have undergone thymectomy with those who seek conventional therapeutic treatment. It has been hypothesised that patients with MG who had thymectomy had a better prognosis.

## Materials and methods

This was a retrospective case-control study carried out in the Department of Medicine and Neurology, Ayub Teaching Hospital, Abbottabad, Pakistan, from October 2020 to September 2021. The purposive sampling technique was used to select MG patients. Clinical data of patients with MG who were being treated at the Neurology and Medicine department of the Ayub Teaching Hospital were evaluated retrospectively. The study was approved by the Institutional Review Board of Ayub Teaching Hospital, Abbottabad, Pakistan (approval number: 5241/ATH).

Inclusion and exclusion criteria

We only included patients who underwent thymectomy at the Ayub Teaching Hospital and control group patients were also selected from the patients who were seeking treatment from the same hospital and had enough clinical information prior to the procedure and follow-up information for at least 12 months afterwards to allow for the evaluation of one-year clinical response. Patients with human immunodeficiency virus (HIV), ocular myasthenia with a normal thymus who have had the condition for less than a year, and patients with recurrent MG were excluded from the study.

Data collection

The medical records of the patients with confirmed MG were evaluated and participants were selected on the basis of the inclusion/exclusion criteria of our study. Matching of controls was done on the basis of sex and age. Acetylcholine receptor antibodies, a positive electromyography (EMG)/repetitive nerve stimulation (RNS) study, and a pyridostigmine test (because edrophonium wasn't accessible) were used to make the diagnosis of MG.

Patients were divided into two groups: Group 1 (case) was the thymectomy MG group (115 patients who had undergone thymectomy) and Group 2 (control) was the MG group (250 patients who were managed without thymectomy). Based on the sampling criteria, 80 patients were excluded, and 20 patients refused to follow up. A total of 96 MG patients were chosen arbitrarily from the sample based on case matching by gender and age.

Thirty-two MG patients with thymectomy (Group 1) and 64 MG patients without thymectomy (Group 2) were selected for investigation. The 32 MG patients with thymectomy were purposively matched with MG controls by age (±1 year) and sex. Two controls were assigned to each MG thymectomy patient. Patients were called to hospital OPD for assessment of treatment outcomes.

Clinical outcomes

Using the criteria of the MGFA [9], disease severity and therapy category were documented at various time intervals. The clinical response was constituted of total stable remission, pharmaceutical remission, and mild manifestation based on the categories of the MGFA post-intervention status (MGFA-PIS) [1-3]. Complete remission is defined as the absence of all MG symptoms for a full year without the need for symptomatic therapy. Remission following therapy is categorized as: (i) Pharmacological and complete stable remission (CSR); this includes Remission CSR in which no clinical weaknesses were found during the evaluation; (ii) Pharmacological remission: similar to CSR, involves taking medication but avoiding cholinesterase inhibitors; (iii) Minimal manifestations (MM): the patient exhibits muscular weakness but no MG symptoms. This class recognises that certain CSR or poor response patients exhibit weakness that is only visible by rigorous examination. MM can be: (a) MM0: went a year without receiving MG therapy; (b) MM1: The patient is still receiving immunosuppression, but no cholinesterase inhibitors or any symptomatic medication; (c) MM2: Takes pyridostigmine 120 mg at a low dosage of cholinesterase inhibitors for at least a year; (d) MM3: Over the past year, received both an immunosuppressant and a cholinesterase inhibitor [10].

MG quality of life 15 (MG-QOL 15) scale

A self-reported questionnaire was developed to assess the quality of life of individuals with MG. The questionnaire comprises 15 items with scores ranging from 0 through 60; the lower the score, the lower the patient's perceived quality of life [11].

Statistical analysis

IBM SPSS Statistics for Windows, Version 26.0 (Released 2019; IBM Corp., Armonk, New York, United States) was used for data analysis. Matched analysis (conditional logistic regression model) was conducted using IBM SPSS Statistics for Windows, Version 20.0 (Released 2011). Odds ratio (RR) estimates are shown as 95% confidence intervals. P< 0.05 was considered significant.

## Results

The final study sample was composed of 32 cases (Group 1: MG patients who underwent thymectomy) and 64 controls (Group 2: MG patients without thymectomy), matched for age and sex. The demographic and clinical characteristics of the sample are shown in Table 1.

**Table 1:** Demographic and clinical characteristics of the cases and controls Mann-Whitney U-test (for continuous variables) and the Chi-squared test or Fisher exact test (for categorical variables) were used to obtain probability values. MGQOL-15: myasthenia gravis quality of life 15; IQR: interquartile range

Variable	Group 1 (Cases)	Group 2 (Controls)	p-value
Male, Female, n (%)	11 (34.3%), 21 (65.6%)	22 (34.3%), 42 (65.7%)	NA
Age (years), mean±SD	35±8.9	37±11.1	0.561
BMI (kg/m^2^)	22.9 ± 3.9	21.1 ± 3.0	0.0001
Associated diseases, n (%)	Yes 17 (53.1%), No 15 (46.8%)	Yes 36 (56.25%) No 28 (43.75%)	0.311
Thymoma, n (%)	24 (22.2%)	5 (35.7%)	0.3173
Dysphagia, n (%)	21 (65%)	49 (76.5%)	0.002
MGQOL-15 score, mean±SD	24.13 ±11.9	20.13 ±21.9	0.005
Plasmapheresis, n (%)	12 (36%)	39 (40%)	0.50
Dose of medication at baseline, median (range)	360 (0–540)mg	370 (110–570)mg	0.183
Dose of medication after one year, median (range)	45 (0–360)mg	370 (30–560)mg	0.004
Mean age at onset (years), (IQR) mean ±SD	36 ±15.4	42 ±12.2	
Mean age at thymectomy (years), (IQR) mean ±SD	42 ±12.4	-	-
Duration (from the beginning of symptoms to thymectomy), mean ±SD	21.6 ±12.4	-	-
Osserman score within one year back I, IIA, IIB, III, IV, n (%)	4 (12.5%), 12 (37.5%), 6 (18.7%), 8 (25%), 2 (6.25%)	8 (13%), 27 (45%), 4 (7%), 12 (3%), 3 (9%)	0.543
Osserman score at last follow-up assessment I, IIA, IIB, III, IV, n (%)	9 (28.1%), 12 (38%), 5 (15.6%), 4 (12.5%), 2 (6.25%)	8 (12.5%), 22 (34.3%), 16 (25%), 8 (12.5%), 10 (15.6%)	0.04

Treatment outcome measures at the last follow-up

**Figure 1:**
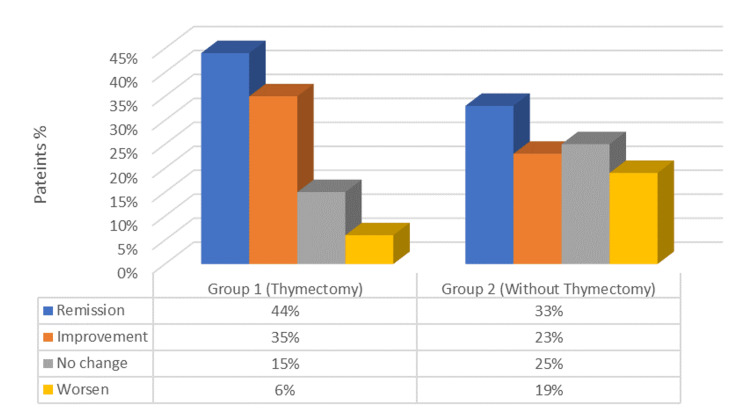
Comparison of long-term outcomes among groups

At the last follow-up visit after a year, 43 patients (45%) were in pharmacological remission. Twenty-six patients (27%) had an MGFA-PIS (post-intervention status) of MM-3, 10 patients (11.5%) had MM-1, 9 patients (8,7%) had MM-2, two patients (1.4%) had MM-0, and six patients (6.25 %) were on complete stable remission (CSR) (Figure 1). Response to thymectomy was overall good with 78.2% of patients having a better response to thymectomy and seven (21.8%) patients having a bad response as shown in (Table 2)

**Table 2:** Response to thymectomy in MG cases with thymectomy (n=32) MGFA-PIS: Myasthenia Gravis Foundation of America post-intervention status

Response to thymectomy, n (%)	
Good Response	25 78%
Poor Response	7 21.8%
MGFA-PIS after Thymectomy	
Remission	14 (43.75%)
Improvement	11 (34%)
No change	5 (15.6%)
Worsening	2 (6.25%)

MGFA clinical classification between groups showed no significant difference (p<0.05). We investigated the relationships between potential variables and the MG prognosis. Dysphagia and low BMI (20 kg/m^2^) were substantially related to poor outcomes. Dysphagia and thymoma were all linked to worse outcomes, according to a single-factor analysis using the Cox proportional hazards model.

There was no statistical significance, although there was a propensity for patients with more than a 3-year evolution to have a poor prognosis (OR 3.01, 95%CI 0.29-5.50, p 0.15) and those with more than a four-year evolution to have a worse prognosis (OR 2.53, 95%CI 0.83-7.7, p 0.06).

In Group 1, being older was linked to a poor prognosis (OR 3.8, 95%CI 1.11-20.32, p 0.04). Patients with more than two years of illness (OR 3.5, 95%CI 0.97-6.39, p 0.08) had a tendency toward a poor prognosis, though this was not statistically significant, whereas cases with more than three years of illness (OR 2.58, 95%CI 0.89-0.6, p 0.04) had a statistically significant poor prognosis. A logistic regression model was used. Age and Osserman stage was discovered to be the two key factors in this model for a poor outcome following thymectomy.

## Discussion

Thymectomy as a treatment for MG results in remissions in a substantial number of cases, allowing for a decrease in the dosage of indicated medication and an improvement in manifestations. There is currently universal agreement that patients with generalized MG who are between the ages of adolescence and 60 years should have surgery. Thymectomy is also recommended for those with severe illness and for people who may have thymoma. In our study, we found that 69% of patients who underwent thymectomy showed good results. The response frequency is consistent with that found in other investigations (Table 3). The responses were 59% in the study by Hatton et al. [12], 71% in the report by Busch et al. [13], and 87% in the study by Frist et al. [14].

**Table 3:** Comparative response to thymectomy in the literature

Author	Study type	Sample	Associations studied	Response
Hatton et al. [11]	Retrospective	52	Patients with generalized myasthenia (IIA, IIB, II, IV) and females responded more favourably.	Remission 27%, Improvement 35%, No change 38%
Busch et al. [12]	Retrospective	86	Patients affected with generalized myasthenia (IIA, IIB, II, IV) responded better.	Remission 19%, Improvement 52%, No change 22%, Worse 7%
Frist et al. [13]	Retrospective	46	Patients younger than 45 years old, females, and those with generalized myasthenia had a favourable prognosis.	Important improvement 63%, Improvement 24%, No change 11%, Worse 2%
Durelli et al. [14]	Retrospective	400	Patients under 30 years of age, females, and those with Osserman I and IIA had a favourable prognosis. Thymoma patients have a dismal prognosis.	Remission 27%, Improvement 57%, No change 11%, Worse 5%
Current study	Retrospective	32	A poor prognosis was associated with patients older than 60 years, Osserman I, long-standing disease prior to surgery, steroid use previous to surgery, large doses of medication prior to surgery, and thymoma or thymic atrophy.	Remission 43.75%, Improvement 34%, No change 15.6%, Worsening 6.25%

The Osserman stage and patient age were shown to be the most important predictive variables in this investigation. Our data indicate that Osserman stage I is associated with a poor response to thymectomy, despite the fact that there is controversy regarding whether patients with stage I (ocular myasthenia) should have thymectomy [9]. On the other hand, the association between advanced age and a substandard response to thymectomy is clear and has been documented in prior research [14,15].

The mean good response in these investigations, which includes the present study, is 76.2 with a 95% confidence interval of 9.5. The length of the disease overall and the time between diagnosis and thymectomy were two additional factors associated with poor outcomes. In a cohort of 317 patients who had thymectomy, Otto and Strugalska found that those with shorter disease durations had a superior response to thymectomy [16]. This was likely due to the fact that a prolonged disease duration creates increased neuromuscular plate damage, which worsens a patient's prognosis. However, thymectomy may allow a dose reduction, as was demonstrated in the MGTX trial, where patients in the thymectomy group had an overall lower prednisone dose and fewer patients were treated with azathioprine. Studies suggest that some form of immunosuppressive therapy is likely to be required in patients with persistent disease activity or clinical relapse after thymectomy [17].

Limitations of the study

This study has significant limitations. First, for MG in general, the description of clinical response requires at least a year without symptoms. Therefore, it is impossible to rule out a selection bias that would remove patients who saw rapid improvement in the first year after thymectomy and a loss of follow-up as a result. At our specialised neuromuscular tertiary care hospital, we believe the majority of patients received treatment and were watched for an adequate amount of time.

Second, the length of follow-up varied widely, and it is anticipated that the relapse rate will increase as the disease develops more slowly. However, this would only strengthen our claim that the initial clinical reaction must be differentiated from the long-term illness course during outcome evaluation. Third, it should be noted that various other variables such as dose, timing, and type of therapy may play an impact, but our research focused on a select number.

## Conclusions

Our findings imply that the current therapeutic approach of patient selection for thymectomy does not result in significantly inferior outcomes for any of the categories that were evaluated. This is the conclusion drawn from our examination of the data. Additionally, we would like to stress how important it is to monitor the long-term illness course independently from the first clinical response. When compared to individuals who have not undergone thymectomy, those who have undergone the procedure have a significant decrease in the amount of anti-myasthenic medication they need to take while experiencing minimal to no aggravation. The thymectomy has been shown to produce significantly superior results. Therefore, thymectomy needs to be made available to AChR-Ab-positive myasthenic patients as a component of their treatment.

## References

[1] Cetin H, Fülöp G, Zach H, Auff E, Zimprich F (2012). Epidemiology of myasthenia gravis in Austria: rising prevalence in an ageing society. Wien Klin Wochenschr.

[2] Rath J, Brunner I, Tomschik M (2020). Frequency and clinical features of treatment-refractory myasthenia gravis. J Neurol.

[3] Phillips LH 2nd (2003). The epidemiology of myasthenia gravis. Ann N Y Acad Sci.

[4] Gilhus NE, Verschuuren JJ (2015). Myasthenia gravis: subgroup classification and therapeutic strategies. Lancet Neurol.

[5] Wolfe GI, Kaminski HJ, Aban IB (2016). Randomized trial of thymectomy in myasthenia gravis. N Engl J Med.

[6] Spillane J, Hayward M, Hirsch NP, Taylor C, Kullmann DM, Howard RS (2013). Thymectomy: role in the treatment of myasthenia gravis. J Neurol.

[7] Wolfe GI, Kaminski HJ, Aban IB (2019). Long-term effect of thymectomy plus prednisone versus prednisone alone in patients with non-thymomatous myasthenia gravis: 2-year extension of the MGTX randomised trial. Lancet Neurol.

[8] Rath J, Taborsky M, Moser B (2022). Short-term and sustained clinical response following thymectomy in patients with myasthenia gravis. Eur J Neurol.

[9] Gilhus NE, Verschuuren JJ (2015). Myasthenia gravis: subgroup classification and therapeutic strategies. Lancet Neurol.

[10] Hehir MK, Hobson-Webb LD, Benatar M (2017). Rituximab as treatment for anti-MuSK myasthenia gravis: multicenter blinded prospective review. Neurology.

[11] Burns TM, Grouse CK, Conaway MR, Sanders DB (2010). Construct and concurrent validation of the MG-QOL15 in the practice setting. Muscle Nerve.

[12] Hatton PD, Diehl JT, Daly BD (1988). Transsternal radical thymectomy for myasthe nia gravis: a 15-year review. Ann Thorac Surg.

[13] Busch C, Machens A, Pichlmeier U, Emskötter T, Izbicki JR (1996). Long-term outcome and quality of life after thymectomy for myasthenia gravis. Ann Surg.

[14] Frist W, Thirumalai S, Doehring C, Merril W, Stewart J, Fenichel G, Bender H (1994). Thymectomy for the myasthenia gravis patient: factors influencing outcome. Ann Thorac Surg.

[15] Durelli L, Maggi G, Casadio C, Ferri R, Rendine S, Bergamini L (1991). Actuarial analysis of the occurrence of remissions following thymectomy for myasthenia gravis in 400 patients. J Neurol Neurosurg Psychiatry.

[16] Otto TJ, Strugalska H (1987). Surgical treatment for myasthenia gravis. Thorax.

[17] Mantegazza R, Bonanno S, Camera G, Antozzi C (2011). Current and emerging therapies for the treatment of myasthenia gravis. Neuropsychiatr Dis Treat.

